# Supplementary material for: Causal Relationship between Aging and Anorexia Nervosa: A White-Matter-Microstructure-Mediated Mendelian Randomization Analysis
Source: Biomedicines. 2024 Aug 16;12(8):1874. doi: 10.3390/biomedicines12081874 (PMC11351342; doi:10.3390/biomedicines12081874)
Supplement: Supplementary file 1 [file biomedicines-12-01874-s001.zip › Supplementary Figure/Figure S4/Singe SNP plot of MR analysis for TL to ubm-b-2035.pdf]

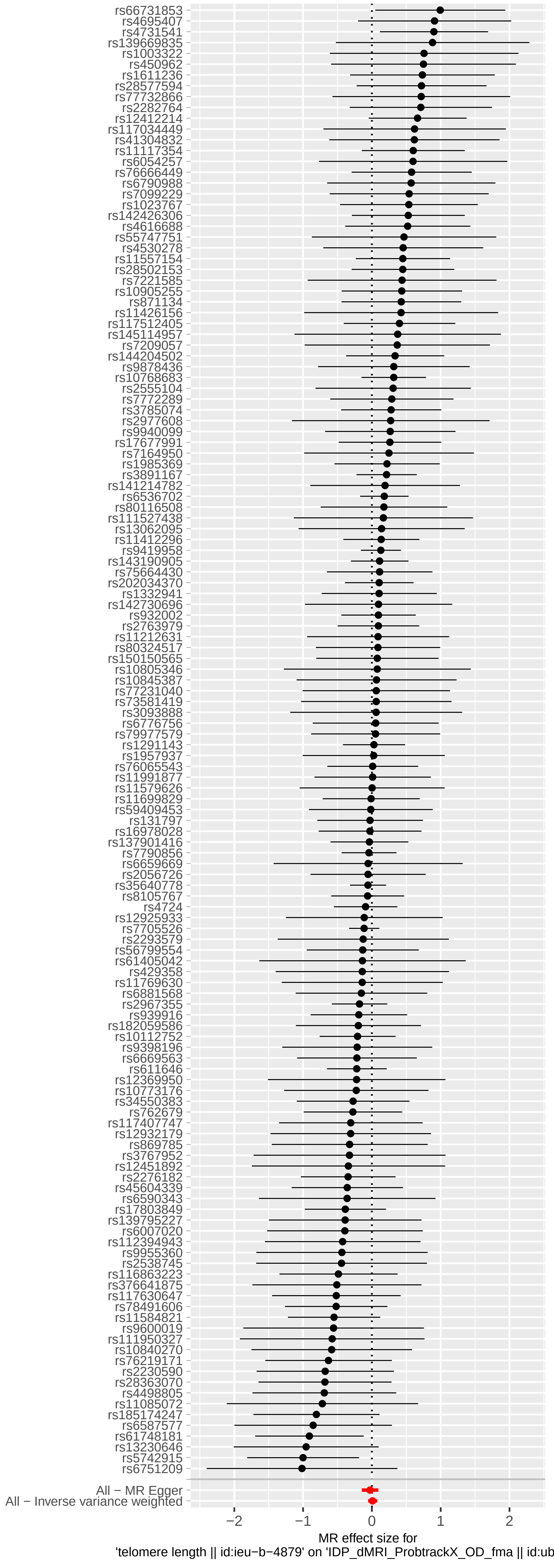

rs66731853
rs4695407
rs4731541
rs139669835
rs1003322
rs450962
rs1611236
rs28577594
rs77732866
rs2282764
rs12412214
rs117034449
rs41304832
rs11117354
rs6054257
rs76666449
rs6790988
rs7099229
rs1023767
rs142426306
rs4616688
rs55747751
rs4530278
rs11557154
rs28502153
rs7221585
rs10905255
rs871134
rs11426156
rs117512405
rs145114957
rs7209057
rs144204502
rs9878436
rs10768683
rs2555104
rs7772289
rs3785074
rs2977608
rs9940099
rs17677991
rs7164950
rs1985369
rs3891167
rs141214782
rs6536702
rs80116508
rs111527438
rs13062095
rs11412296
rs9419958
rs143190905
rs75664430
rs202034370
rs1332941
rs142730696
rs932002
rs2763979
rs11212631
rs80324517
rs150150565
rs10805346
rs10845387
rs77231040
rs73581419
rs3093888
rs6776756
rs79977579
rs1291143
rs1957937
rs76065543
rs11991877
rs11579626
rs11699829
rs59409453
rs131797
rs16978028
rs137901416
rs7790856
rs6659669
rs2056726
rs35640778
rs8105767
rs4724
rs12925933
rs7705526
rs2293579
rs56799554
rs61405042
rs429358
rs11769630
rs6881568
rs2967355
rs939916
rs182059586
rs10112752
rs9398196
rs6669563
rs611646
rs12369950
rs10773176
rs34550383
rs762679
rs117407747
rs12932179
rs869785
rs3767952
rs12451892
rs2276182
rs45604339
rs6590343
rs17803849
rs139795227
rs6007020
rs112394943
rs9955360
rs2538745
rs116863223
rs376641875
rs117630647
rs78491606
rs11584821
rs9600019
rs111950327
rs10840270
rs76219171
rs2230590
rs28363070
rs4498805
rs11085072
rs185174247
rs6587577
rs61748181
rs13230646
rs5742915
rs6751209
All − MR Egger
All − Inverse variance weighted
−2
−1
0
1
2
MR effect size for
'telomere length || id:ieu−b−4879' on 'IDP_dMRI_ProbtrackX_OD_fma || id:ub
